# NIA Priorities on Emotional Well-Being

**DOI:** 10.1093/geroni/igab046.784

**Published:** 2021-12-17

**Authors:** Janine Simmons

**Affiliations:** NIA, Bethesda, Maryland, United States

## Abstract

In 2021, NIH funded six high-priority research networks designed to develop resources to support and advance the study of emotional well-being (EWB) and its core components. These research networks aim to advance the field by facilitating transdisciplinary research in the social, behavioral, psychological, biological, and neurobiological sciences. The National Institute on Aging (NIA) co-sponsored the RFA, and provided funding for NEW Brain Aging, because of the central importance of EWB to health trajectories across the adult lifespan. In this presentation, Dr. Simmons, Chief of the Individual Behavioral Processes Branch within the NIA Division of Behavioral and Social Research (BSR), will discuss how EWB research fits within NIA priorities. She will then facilitate open discussion about NIA and BSR’s vision for the EWB ‘network of networks,’ the synergy of NEW Brain Aging with other members of the larger network, and the opportunities these networks will provide for investigators interested in EWB.

